# Personalizing Therapy Outcomes through Mitogen-Activated Protein Kinase Pathway Inhibition in Non-Small Cell Lung Cancer

**DOI:** 10.3390/biomedicines12071489

**Published:** 2024-07-05

**Authors:** Hasan Alsharoh, Paul Chiroi, Ekaterina Isachesku, Radu Andrei Tanasa, Ovidiu-Laurean Pop, Radu Pirlog, Ioana Berindan-Neagoe

**Affiliations:** 1 Research Center for Functional Genomics, Biomedicine and Translational Medicine, “Iuliu Hatieganu” University of Medicine and Pharmacy, 400337 Cluj-Napoca, Romania; hasan.alsharoh@elearn.umfcluj.ro (H.A.); chiroi.paul@umfcluj.ro (P.C.); ekaterina.isachesku@umfcluj.ro (E.I.); ioana.neagoe@umfcluj.ro (I.B.-N.); 2 Panomics, Inc., 228 Park Ave S, PMB 22322, New York, NY 10003, USA; radu@panomics.bio; 3 Department of Morphology Sciences, University of Oradea, 410087 Oradea, Romania; popo@uoradea.ro

**Keywords:** non-small cell lung cancer, mitogen-activated protein kinase, personalized medicine, cancer research, anti-cancer therapy, targeted therapy

## Abstract

Lung cancer (LC) is a highly invasive malignancy and the leading cause of cancer-related deaths, with non-small cell lung cancer (NSCLC) as its most prevalent histological subtype. Despite all breakthroughs achieved in drug development, the prognosis of NSCLC remains poor. The mitogen-activated protein kinase signaling cascade (MAPKC) is a complex network of interacting molecules that can drive oncogenesis, cancer progression, and drug resistance when dysregulated. Over the past decades, MAPKC components have been used to design MAPKC inhibitors (MAPKCIs), which have shown varying efficacy in treating NSCLC. Thus, recent studies support the potential clinical use of MAPKCIs, especially in combination with other therapeutic approaches. This article provides an overview of the MAPKC and its inhibitors in the clinical management of NSCLC. It addresses the gaps in the current literature on different combinations of selective inhibitors while suggesting two particular therapy approaches to be researched in NSCLC: parallel and aggregate targeting of the MAPKC. This work also provides suggestions that could serve as a potential guideline to aid future research in MAPKCIs to optimize clinical outcomes in NSCLC.

## 1. Introduction

Lung cancer (LC) is a highly invasive malignancy that is responsible for most cancer-related deaths, according to the global cancer statistics for 2020 [1]. Non-small cell lung cancer (NSCLC) accounts for up to 80% of all LC cases and has a five-year survival rate of 27% [1,2,3]. Despite the cutting-edge developments in treatment modalities, patient prognosis remains poor [4]. The low survival rate is primarily attributed to the complex molecular heterogeneity within NSCLC tumors, late detection, and treatment resistance [5].

The mitogen-activated protein kinase cascade (MAPKC) is a complex network of interacting molecules implicated in cellular functions such as growth, differentiation, inflammation, survival, and apoptosis [6]. Defects in the MAPKC are often implicated in oncogenesis, disease progression, and drug resistance in cancer and other diseases [6,7]. To optimize personalized anti-cancer therapies, MAPKC-related drug targets have been used to design MAPKC inhibitors (MAPKCIs), which have shown varying efficacy in treating NSCLC and other cancers [8,9].

While MAPKCIs have been under investigation for over 20 years, many inhibitors have not gained approval in NSCLC, as their in vitro efficacy did not translate into clinical trials [10]. This is likely due to the low selectivity of tyrosine kinase inhibitors (TKIs), as kinases often have similar drug pockets, leading to increased toxicity and the clinical failure of many MAPKCIs [11]. The most frequent MAPKC-associated driver gene mutation involved in NSCLC is the *BRAF*^V600E^ mutation [12]. MAPKCIs targeting *BRAF* in NSCLC have shown promising results, leading researchers to examine the possibility of targeting other components of the MAPKC [13].

The inconsistent efficacy of MAPKCIs has been associated with increasing tumor mutational burden (TMB) and acquired treatment resistance [14]. The presence of many possible driver gene mutations related to the MAPKC allows tumors to circumvent mitogen-activated protein kinase (MAPK) pathway inhibition and resume proliferation [15]. In addition, the toxicity profiles of older MAPKCIs lead to a reduced interest in investigating the inhibition of many MAPKC components. Still, with recent advances and numerous patents registered for highly selective MAPKCIs, this might change shortly [10]. Moreover, the low mutation rate of other MAPKC components may have shifted the focus to the commonly mutated upstream regulators of the MAPKC, such as *KRAS*, *EGFR*, *MET*, *ROS1*, *ALK*, and *RET* [12]. However, recent studies have shown that multiple downstream components of the MAPKC could serve as helpful drug targets, especially when combined with other therapeutic approaches, such as immunotherapy [16,17].

Since upstream and downstream components are becoming more accessible to targeting by novel MAPKCIs, it is essential to discuss future directions based on recent evidence in personalized medicine [18]. In this review, we provide a brief overview of the MAPKC and some of the inhibitors used to target this pathway in NSCLC while addressing gaps in improving the therapeutic efficacy of the MAPKC. Mainly, we characterize parallel and aggregate targeting of the MAPKC as systematic combinatorial treatment approaches targeting the MAPKC.

These approaches could potentially improve treatment outcomes in NSCLC patients with treatment-induced mutations. Parallel and aggregate targeting of the MAPKC have been shown to overcome treatment resistance and improve therapeutic efficacy in specific contexts in patients with various cancers [19,20]. An example of the parallel targeting approach is currently studied in glioblastoma, using dual inhibition of the MEK/ERK pathway through ralimetinb and the p38 MAPK pathway through binimetinib [21]. A commonly used example of aggregate targeting is dabrafenib and trametinib combinations to treat *BRAF*-mutated NSCLC, targeting RAF and MEK1/2 [22]. These approaches may prove beneficial for many patients as we move towards personalized therapy.

As novel compounds are used to target more components of the MAPKC, such as HWY336 inhibiting MKK4/7 [23], and pyrazolo[3,4-d]pyrimidines as MKK3 inhibitors [24], parallel and aggregate targeting could be utilized to achieve optimal treatment response to these compounds. Further, as increasing reports have suggested that assessing MAPKC activity through phosphorylated ERK had proved inaccurate [25,26], more accurate modalities should be utilized in assessing the activity of the MAPKC when using parallel and aggregate targeting. Thus, this narrative literature review provides essential insights into the potential treatment methods for future research and discusses alternative approaches to assess MAPKC activity more accurately, both in preclinical and in clinical trials.

## 2. An Overview of the MAPKC and Its Components

The MAPKC has been demonstrated to transmit extracellular and intracellular signals [27]. The pathways within the MAPKC control the transduction of various stimuli into suitable physiological reactions. These outcomes include proliferation, differentiation, cellular development, inflammation, and apoptosis [28,29]. The activation of the MAPKC starts with extracellular stimuli that trigger the activation of tyrosine kinase receptors (TKRs). These stimuli may include one or several growth factors, hormones, cytokines, or the intracellular molecules–TKRs interaction that activates the MAPKC [30,31].

Following the stimulation of TKRs, signals from TRK to MAPK elements are transduced through several intermediate components. In NSCLC, for example, when the epidermal growth factor (EGF) binds to its receptor (EGFR), the growth factor receptor-bound protein 2 (GRB2) establishes stable complexes with the tyrosine phosphorylated EGFR [32]. This leads to the activation of downstream pathways through an exchange protein, including the son of sevenless (SOS), which activates proteins from the RAS superfamily, including small GTPases such as HRAS, KRAS, and NRAS [33]. Other stimuli that are transduced to affect downstream MAPKC components include interleukin (IL)-1, tumor necrosis factor-alpha (TNF-a), and reactive oxygen species (ROS) [34,35].

From this point, the MAPKC starts with several proteins called MAPK kinase kinases (MAP3Ks). MAP3Ks are mainly represented by the Raf oncoproteins [36]. Other MAP3Ks include mixed-lineage kinases (MLKs), apoptosis signal-regulating kinases (ASKs), zipper sterile-alpha-motif kinase (ZAK), and TAK1 [34]. Subsequently, the cascade activates MAPK kinases (MAP2Ks), including MAP kinase-ERK kinase (MEK) 1/2 isoforms, and MAP kinase kinase (MKK) 3/4/6/7 isoforms [34]. This ultimately drives the progression of downstream pathways by phosphorylating MAPKs and elevating their enzymatic actions [37]. The most notable MAPK families that have been identified so far include c-Jun N-terminal kinase (JNK) (including the JNK1/2/3 isoforms), p38 MAPKs (including the p38α/β/γ/δ isoforms), and ERK (including the ERK1/2 isoforms) [29].

MAP3Ks are recently gaining attention regarding their functions and relevance in determining cell fate and the functional regulation of downstream MAPKC components [34]. The mammalian MLK subgroup consists of four members (MLK1-4), each implicated in various functions. Notably, MLK3 is involved in activating several MAP2K members, primarily MKK7, and to some degree MKK4, depending on the extent of TNF-a-induced JNK activation [38,39]. Of the ASKs, ASK1 is activated by various stimuli, including TNF-a-generated ROS, leading to the regulation of JNK through MKK4 and MKK7 and p38 MAPK through MKK3 and MKK6 [35]. ZAK regulates JNK and p38 MAPK similarly to ASK1 and has been implicated in lung cancer development [40]. Recent reports indicated that ZAK may play an essential role in cell migratory functions in colorectal cancer cell lines by affecting the ERK pathway as well [41]. IL-1-activated TAK1 is also implicated in transcriptional and RNA-targeted mechanisms of gene regulation by playing a part in JNK and p38 MAPK phosphorylation through MKK4 and MKK3/6, respectively [42].

The functions of MAP2Ks have been studied more thoroughly than MAP3Ks, with MEK1/2 being one of the most commonly targeted MAPKC components due to its implication in transducing signals through the RAF/MEK/ERK pathway [43]. MEK1/2 are essential components in the phosphorylation of downstream ERK1/2, with ERK being able to retrophosphorylate MEK1 through feedback loops [44]. In the context of TNF-a-induced MAP3K activation, MKK7 is essential in activating JNK isoforms, while MKK4 activity represents the extent of JNK activation [39]. While MKK4 also plays a role in JNK and p38 MAPK regulation, MKK3/6 are specific to the p38 MAPK pathway [45].

Despite extensive studies, the functions of MAPKs have yet to be fully characterized. ERK1/2 signaling is thought to regulate Bcl-2 proteins and promote tumor survival [27]. However, the complexity of the mechanisms regulated by ERK1/2 and its involvement in many cellular regulatory signaling pathways make it challenging to elucidate the functions of these MAPKs [46]. While studies have generally agreed on the pro-carcinogenic effects of ERKs, the literature also attributes pro-apoptotic functions to this pathway [47,48].

JNK signaling is also implicated in oncogenesis, although some studies provided evidence that JNK could act as a tumor suppressor, depending on the context [49,50]. It was observed that in normal epithelium, JNK acts as a tumor suppressor, and that in tumors with hyperactive Ras-signaling, EGFR activation leads to a change in JNK functioning through Ras-mediated switching, inducing tumor growth in *Drosophila* [51]. In contrast, a study by Itah et al. identified JNK deficiency as involved in developing HER2^+^ breast cancer in mice. The authors attributed the proliferation mechanism to a JNK-deficiency-related increase in integrin α6β4 that would bind to HER2 and amplify its signaling, leading to an elevation in the TMB [52].

P38 MAPKs are also implicated in various functions and were initially defined as tumor suppressors, with recent evidence highlighting their potential to promote tumor proliferation [53]. Additionally, activation of p38 MAPKs induces chemoresistance in LC [6]. Interestingly, one novel compound was found to suppress tumor proliferation in *EGFR*-mutated NSCLC through activating JNK/p38 MAPK pathways, indicating the variable functionality of these pathways [54].

**Figure 1:**
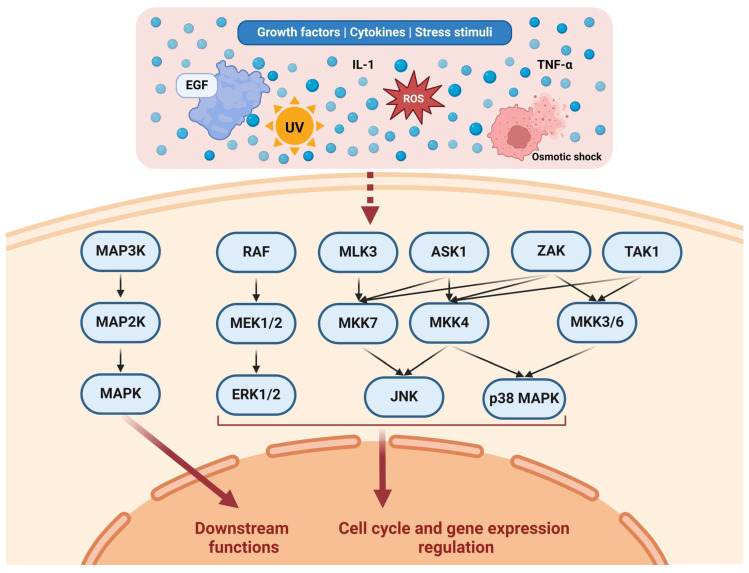
Representation of the MAPKC. Various stimuli affect different upstream pathways. For example, EGF affects the RAF/MEK/ERK pathway through EGFR/RAS/RAF [32,33]. MAP3Ks constitute several kinases, including RAF, MLK3, ASK1, ZAK, and TAK1. MAP3Ks regulate a number of MAP2Ks through complex activation patterns. MAP2Ks constitute multiple kinases as well, including MEK1/2, MKK7, MKK4, and MKK3/6. MAP2Ks regulate MAPKs, including ERK1/2, JNK, and p38 MAPK. Collectively, the MAPKs regulate downstream functions relevant to the cell cycle, cell functions (such as adhesion and migration), and gene expression. Created with Biorender.com https://www.biorender.com/ (accessed on 1 July 2024).

Ultimately, MAPKs transduce signals to the nucleus in various ways. For example, ERKs 1 and 2 were shown to directly bind to DNA and repress the transcriptional functions of several genes [55]. It is estimated that ERK1/2 can phosphorylate hundreds of proteins involved in diverse cellular functions. These substrates include transcription factors, kinases, cytoskeletal proteins, and enzymes [56]. The JNK and p38 MAPK effects were observed to be based mainly on regulating several nuclear components [57]. Figure 1 illustrates various pathways in the MAPKC.

While regulators of the MAPKC, such as EGFR and KRAS, are commonly altered in cancer, mutations of MAPKs are rarely observed in cancer patients [58,59]. Many known mutations in MAPKs have only been generated in vitro or in vivo using murine models for reasons that remain to be elucidated [17]. It is difficult to attribute the low incidence of mutations in this pathway to any reason due to the lack of proper pathway characterization [53,60,61]. Considering all this, future studies should further investigate the mechanisms through which MAPKs preserve a low rate of mutations.

## 3. MAPKC Inhibitors Investigated in Preclinical Studies

As the MAPKC accumulates many mutations that could contribute to oncogenic signaling, it presents an attractive topic for drug development and molecular targeting approaches. Since the MAPK pathway is a complex signaling cascade involved in the regulation of various cellular processes, including cell proliferation, differentiation, and survival, it becomes evident that different MAPKCIs, which can target various components of the pathway, will disrupt signaling and alter cellular behavior in a specific way, depending on the localization of MAPK components within the cascade where the inhibition starts. This means that inhibitors targeting upstream elements of the pathway, such as TKRs or, more specifically, RAF kinases, can block signal transmission from extracellular stimuli to the MAPK cascade. For example, inhibitors like gefitinib [62] and erlotinib [63,64] selectively target EGFR, inhibiting its kinase activity and downstream signaling through the RAF-MEK-ERK pathway. Moving downstream, MEK inhibitors such as trametinib [65] and cobimetinib [66] target MEK1/2, preventing the phosphorylation and activation of ERK1/2. Finally, inhibitors targeting the latest elements within the pathway, such as ERK inhibitors, directly block the activity of ERK1/2. Examples include ulixertinib [67], which inhibits ERK phosphorylation and downstream signaling. Overall, the diverse array of MAPK inhibitors provides targeted therapeutic options for disrupting aberrant MAPK signaling in cancer and other diseases.

MAPKCIs are frequently used to target MAPKC kinases, in addition to other pathways. Dabrafenib is a mitogen-activated protein kinase signaling cascade inhibitor (MAPKCI) shown to induce an apoptotic response through BRAF inhibition, reducing ERK1 and 2 phosphorylation [68,69]. The combination was shown to have low pro-apoptotic responses in specific samples in an in vitro study, particularly in tumors with a silenced *Rbms3* gene, warranting further investigation [69]. Importantly, one study assessed the effects of dabrafenib on a gefitinib-resistant NSCLC cell line (through an *EGFR* mutation) and found a reduction of around 30–45% in cell growth [70]. Another example of a BRAF inhibitor is vemurafenib, a targeted agent with proven efficacy in inducing apoptosis in *BRAF*-mutated NSCLC cell lines [71]. Interestingly, a study showed that DNA damage conferred by vemurafenib resensitized chemotherapy-resistant NSCLC cell lines to chemotherapy, likely through acting on ERK1 and 2 [72].

Binimetinib, an important MEK/ERK inhibitor, was observed to induce autophagy, apoptosis, and growth inhibition in NSCLC cell lines through G1 cell cycle arrest [73]. Interestingly, binimetinib facilitated Akt activation while inhibiting ERK signaling, which could potentially cause a paradoxical proliferative response [73,74]. However, combining binimetinib with a PI3K inhibitor reduced Akt activation and demonstrated a more potent apoptotic response [73].

Cobimetinib, an MEK inhibitor, has been shown to reduce ERK phosphorylation, albeit only inducing anti-proliferative responses in NSCLC cell lines, without apoptotic activity [75]. An in vitro study showed that cobimetinib monotherapy increased Akt signaling, which is associated with a poor prognosis in NSCLC patients, while combining cobimetinib with a PI3K/mTOR inhibitor significantly reduced Akt signaling [75]. Cobimetinib has shown different results in vitro, with one study suggesting that different regulators affected cobimetinib response, confirming the hypothesis through *MAPK7* knockdown, leading to an improved treatment response and reducing resistance [76]. Another study further elaborated that while combinations in vitro successfully circumvent treatment resistance, clinical studies showed underwhelming results due to reduced gene expression of MEK/PI3K pathway regulators, leading to a decreased apoptotic response in tumor cells [77]. However, a clinical study found that a combination of cobimetinib and an ERK inhibitor resulted in a stable disease response in 29% of patients with different metastatic solid tumors, including three NSCLC patients with the *BRAF* mutation [78]. Regardless, the trial was terminated due to the overlapping and cumulative toxicity of the combination, yet the authors conclude that these results do not preclude further evaluation of concurrent BRAF and MEK inhibition [78].

Trametinib is another MEK inhibitor shown to induce a paradoxical proliferative response in KRAS-mutant NSCLC cells as monotherapy by increasing mitochondrial oxidative phosphorylation [79]. Notably, trametinib and a mitochondrial complex inhibitor successfully arrested tumor growth and circumvented trametinib resistance [79]. This potential targeting of parallel pathways has been evidenced to produce potentiated responses [69,76]. Contrary to cobimetinib, trametinib in vitro findings translated well into in vivo studies; one recent example is a study combining trametinib and a LARS1 inhibitor (BC-LI-0186) that led to a synergistic antitumor effect [80]. This effect was due to the paradoxical activation of the MAPK pathway due to BC-LI-0186 mTORC1 inhibition, alleviated by trametinib [80].

Pimasertib was shown to target both MEK 1 and 2, decreasing ERK 1 and 2 phosphorylation in NSCLC cell lines [81]. Surprisingly, combining rigosertib with a murine double minute 2 (MDM2) inhibitor resulted in a synergistic activity that induced apoptosis in NSCLC cells, although the results were limited by acquired p53 mutations [81]. P53 mutations are often associated with drug resistance, affecting treatment efficacy [82].

SB202190 is a p38 MAPKCI, which was shown to have further activity on p53 and ROS1 signaling, attenuating apoptosis in NSCLC cell lines [83]. Interestingly, SB202190 was also shown to reduce the phosphorylation of ERK and Akt simultaneously to p38, showing a complex range of activity [83]. Interestingly, ROS1-TKI treatment deprivation was found to cause increased ROS1 signaling and an upregulated p38 MAPK pathway [84]. Treatment with SB202190 partially rescued cell viability through p38 MAPK inhibition, showing the contextual relevance of the p38 MAPK pathway in apoptosis [84]. Few studies have documented this compound’s effects on the MAPKC in NSCLC.

While treatment resistance often impedes the efficacy of MAPKCIs, several compounds have been found to resensitize cell lines to other therapies. One example is Asiatic acid, an anti-cancer drug shown to suppress tumor growth by interacting with many pathways, including the JNK and p38 MAPK pathways [85]. Treating cisplatin-resistant NSCLC cells with Asiatic acid resensitized the cell lines to cisplatin through downregulating MALAT1, a long non-coding RNA (lncRNA) associated with chemoresistance [86]. Asiatic acid has also been confirmed to interact with microRNAs (miRNAs), including miRNAs affecting BCL2, directly and indirectly affecting p38 MAPK and cell apoptosis, warranting further research into Asiatic acid in NSCLC [87].

**Figure 2:**
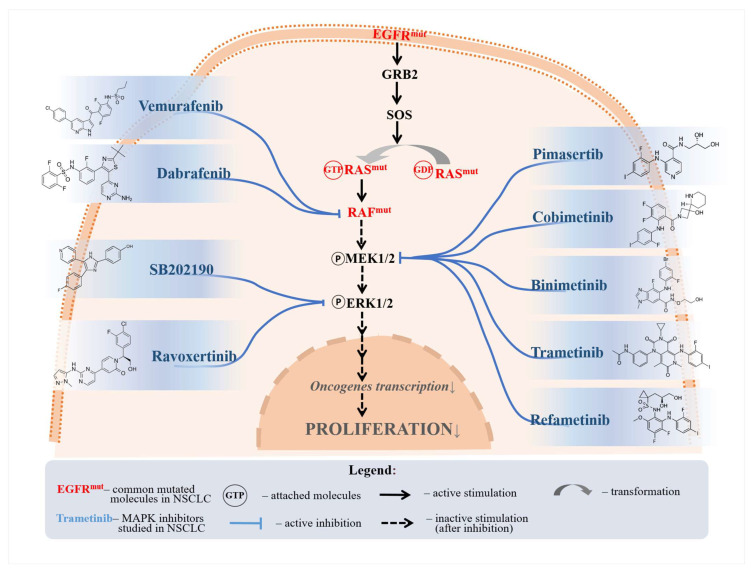
A selection of MAPKC inhibitors and their respective inhibited molecules. Vemurafenib and dabrafenib were shown to inhibit proliferation through RAF inhibition. Pimasertib, cobimetinib, binimetinib, trametinib, and refametinib were all shown to reduce MEK phosphorylation, thus inhibiting NSCLC proliferation. SB202190 and ravoxertinib were shown to affect ERK signaling.

Losmapimod is another p38 MAPKCI, which was demonstrated to resensitize gefitinib-resistant NSCLC cell lines to gefitinib in vitro [88]. Losmapimod was shown to confer its effects by downregulating the phosphorylation of STAT3, p21, and cyclin D1 proteins [88]. Downregulation of STAT3 was found to induce apoptosis and G1 arrest in esophageal carcinoma cells. In contrast, the downregulation of p21 and cyclin D1 shows proliferation suppression and chemosensitivity in lymphoma cell lines [89,90]. Nonetheless, these findings were not validated in NSCLC. Figure 2 illustrates the pathways through which several selected drugs primarily affect the MAPKC.

A selection of the described studies is found in Table 1. Although there is a large body of literature regarding MAPKCIs, the studies described were selected as examples of specific contexts in NSCLC cell lines where MAPKCIs would be used to circumvent treatment resistance, cause paradoxical effects that would be prevented when combined with other treatments, or alter cell fate indirectly through different pathways. The studies provide an overview of the improved efficacy of MAPKCIs when in combination with other treatment modalities and examine the molecular targets of MAPKCIs in NSCLC. These examples also indicate the most commonly used modalities to assess MAPKC activity by phosphorylating MAPKC components.

While these works also show outliers of treatment responses, it is imperative to note that currently, one of the most clinically successful MAPKCI combinations is the BRAF/MEK inhibition of dabrafenib/trametinib, with a 63–34% overall response rate in metastatic LC patients [100]. An indication of the reasons for the large patient cohort not responding to treatments would fall into the outlier circumstances, and further in vitro assessments should provide a perspective towards these reasons. Other MAPKCI combinations have not yet found a similarly high response rate, likely because it takes specific activation patterns of upstream and downstream MAPKC components to induce cell-fate changes in tumors [34]. This also urges future studies to use systematic approaches of combining MAPKCIs and the appropriate evaluation of MAPKC component activities. An elaborate investigation of the effects of MAPKCIs through systematically designed combinations on cell fate using network analysis approaches would provide more accurate insights in vitro, likely helping implement more clinically successful combinations.

Despite in vitro experiments thoroughly evaluating MAPKCIs, assessing alterations in gene expression for genes encoding MAPKC components in NSCLC cell lines following treatment with MAPKCIs remains a rare modality of evaluating treatment efficacy. The phosphorylation of markers such as ERK, used by most studies to assess the efficacy of MAPKCIs, has recently been insufficient in depicting the molecular activity of the MAPKC [25]. This was attributed to negative feedback loops elicited by ERK [101]. Elaborating genetic alterations before and following treatment could provide a more thorough understanding of the variation in response and sensitivity to these targeted therapies [102]. Evidence from in vitro studies in other cancers suggests that mutations in MAPKC components could affect treatment response and potentially lead to pro-tumor activity [103,104].

Alterations incurred in the MAPKC are not only elicited by the components of the MAPKC but also by associated non-coding RNAs (ncRNAs) [105]. Various investigations revealed that several categories of ncRNAs were affected by changes in the MAPKC, including miRNAs and lncRNAs [105,106,107]. Moreover, ncRNAs can also regulate the MAPKC, making them an attractive target to be characterized in other future studies [106]. For example, MALAT1 is an MAPKC-related lncRNA that is upregulated in lung cancer while implicated in drug resistance and tumor proliferation, as it promotes DNA repair in cancer cells [86,108]. To achieve this, MALAT1 sponges miR-146a and miR-216b to protect *BRCA1*, an essential factor in DNA repair [108]. Although the literature indicated the importance of ncRNAs in regulating the MAPKC, research assessing MAPKC-associated ncRNA expression alterations following MAPKCI treatment is scarce [5].

## 4. The Use of MAPKCIs When Translated to Clinical Trials

Many MAPKCIs have been patented in recent years, as their target profile is becoming more selective due to the current developments [10]. Therefore, with in vitro studies on MAPKCIs showing promising results, many of these compounds are being tested in several clinical trials, both as monotherapies and in combination with other treatments. Trametinib and dabrafenib combinations demonstrated a response rate of 68.4% in one clinical trial (NCT01336634) in a *BRAF^V600E^*-mutant metastatic NSCLC sample [109]. Notably, this trial characterized the genomic alterations in the studied sample through NGS and reported that the most extended progression-free survival was achieved in tumors harboring *BRAF* or *KRAS* mutations. The authors also reported decreased survival in those with co-occurring BRAF and PI3K pathway mutations. A trametinib and dabrafenib combination had a higher response rate than dabrafenib monotherapy, reported earlier at around 33% [110]. These results show the importance of utilizing combination therapies to address mutations using systematic approaches, taking advantage of the genomic landscape of targeted tumors.

Another trial (NCT02276027) reported an overall response rate (9.1%) in advanced NSCLC patients treated with binimetinib monotherapy, with tumors harboring *KRAS*, *NRAS*, or *BRAF* mutations not showing satisfactory responses. Moreover, the authors reported that progressive disease was the most common reason for treatment discontinuation (63.6%). While the study used genetic mutations to guide treatment, it did not conduct further analyses on other genetic markers beyond the ones used for treatment initiation, attributing this to fewer patients [111]. It is well known that monotherapy using MEK inhibitors has not been promising as of yet [43]. This further highlights the need for better combinational therapies involving MEK inhibition with higher clinical efficacy.

While the ERK1 and 2 inhibitor ravoxertinib lacks in vitro studies on NSCLC, it was evaluated in combination with cobimetinib in a phase I study (NCT02457793) in patients with locally advanced or metastatic solid tumors, including NSCLC [78]. However, the study reported poor response and a high toxicity profile elicited by the combination, which resulted in study termination. A trial (NCT01988896) suggested a lack of clear benefits when combining cobimetinib with an immune checkpoint inhibitor over PD-L1 inhibitor monotherapy [112]. As post-treatment genomic assessment and testing for MAPKC alterations have not been performed, a precise mechanism of why this lack of benefit is occurring is not yet understood.

In a clinical trial (NCT01390818) investigating the maximum tolerated dose of pimasertib in combination with a PI3K/mTOR inhibitor, progressive disease occurred in 46% of the study sample (146 patients with advanced solid tumors) [113]. Surprisingly, the study reported a response rate of 5% in the NSCLC cohort, which harbored *KRAS* or *NRAS* mutations [113]. Therefore, the phase I trial concluded that parallel inhibition of MAPK and PI3K/mTOR is not tolerable long term. Due to the low treatment response, correlations between the treatment’s clinical activity and mutations in the sample were not assessed.

A dose-escalating trial (NCT00785226) using the potent MEK1/2 inhibitor refametinib combined with a Raf-1/BRAF multi-kinase inhibitor has provided evidence that this combination effectively reduced ERK phosphorylation [114]. While the study included a low sample of patients with various solid tumors, with only one NSCLC patient, the authors reported that stable disease was achieved in 42.1% of the population for 15 weeks or more [114]. These studies show the potential of using combinations of MAPKCIs to target mutations in vulnerable NSCLC patient subsets and the importance of identifying novel combinations. Table 2 summarizes the discussed examples of recently completed clinical trials on examples of MAPKCIs in NSCLC patients.

While several clinical trials have assessed MAPKCIs in NSCLC, most studies focused on late-stage NSCLC, which has been reported to incur mutations that may decrease the treatment response of MAPKCIs [115]. More importantly, many clinical trials documenting study efficacy for MAPKCIs have reported low accrual, limiting the statistical power of their analyses, which does not allow any conclusions to be drawn from their results [109,111,114].

Furthermore, current clinical trials should perform evaluations of the genomic and transcriptomic changes of MAPKC targets following the administration of MAPKCI treatments. Recent reports revealed that assessments of the gene targets of the MAPKC would allow for a more accurate evaluation of the activity of the MAPKC [25]. Clinical trials continue to assess MAPKCI activity through phosphorylation of the pathway’s components, as evidenced by in vitro studies, which provide an inaccurate evaluation [25,114].

Moreover, an aspect that should be considered is the advanced tumor stage in NSCLC patients enrolled in clinical trials investigating MAPKCIs. It is established that the advanced tumor stage is associated with high tumor mutational burden, likely contributing to TKI-desensitizing mutations [116,117]. The complexity of the MAPKC and its interplay and crosstalk with other pathways might be considered for future trials, as treating NSCLC based on a single mutation may not be sufficient to ensure TKI efficacy [118].

## 5. Potential Strategies to Improve MAPKCI Response and Overcome Treatment Resistance

### 5.1. Parallel Targeting of the MAPKC

As previously described, the MAPKC consists of three “layers” of kinase networks, MAP3Ks, MAP2Ks, and MAPKs [36], with each of these layers primarily composed of three interconnecting parallel pathways, RAF/MEKKs/MLKs representing the MAP3K layer, MEK/MKKs(3/6)/MKKs(4/7) MAP2Ks, and ERK/p38/JNKs for the MAPK layer [28]. In this review, we define parallel targeting of the MAPKC as using any combination of selective MAPKCIs that may target multiple kinases across the parallel pathways within the MAPKC. This characterization is significant, as each MAPKC layer may have been implicated in distinct functions requiring inhibition on multiple pathways to elicit a therapeutic response [119]. While previous research established that RAF activates ERK1/2 downstream, recent literature indicated that the regulation of upstream targets with downstream MAPKC components is more complex than initially thought [120]. For example, it was shown that RAFs and ASKs (MAP3Ks) preferentially activate JNK and p38 to a greater extent than how ZAK and TAK1 (MAP3Ks) would activate JNK and p38 [34]. This distinction in activation patterns and their effects on downstream targets indicates the importance of systematically targeting these layers across multiple pathways.

In vitro research found that simultaneous knockdown of the parallel MAP3K pathways, particularly of TAK1, MEKK2, and RAF1, suppressed cellular proliferation in HeLa cells [121]. Inhibition of MAP3Ks was further shown to suppress viability in RAS-mutant lung cancer cell lines exposed to hyperthermia but not in wild-type cell lines, and mutations in MAP3K were shown to induce resistance in multiple cancers [122,123]. Therefore, it is suggested that parallel inhibition of the MAP3Ks may reduce treatment resistance when treatment is tailored to patients’ mutational profiles [124].

As parallel targeting of the MAP3Ks is relatively novel, with standard practice often combining MAPKCIs with other pathways’ inhibitors, such as mTOR inhibitors, the literature lacks documented assessments of parallel inhibition of the MAP2Ks and MAPKs in NSCLC [125]. Several compounds with highly selective activity towards MEKs and MKKs have been identified and showed promising preliminary results. However, they have not yet been assessed in NSCLC [126,127,128,129]. The literature suggests that MKK3 is involved in chemotherapy resistance, so assessing the effects of MAPKCIs on treatment response in combination with chemotherapy is imperative.

**Figure 3:**
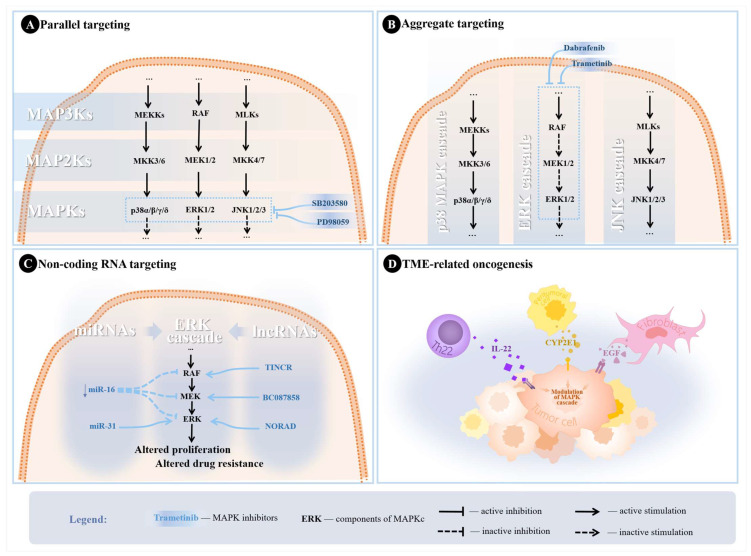
Potential strategies utilized in the literature to overcome treatment resistance. (**A**) Parallel targeting of MAPKs through SB203580 (p38 MAPK pathway inhibitor) and PD98059 (ERK pathway inhibitor). (**B**) Aggregate targeting of ERK cascade with trametinib and dabrafenib for MEK and RAF inhibition. (**C**) Scheme depicting examples of ncRNAs modulating the ERK cascade in NSCLC leading to altered tumor proliferation and treatment resistance. Downregulation of miR-16 was associated with hyperactive MEK/ERK pathways, while miR-31 promotes proliferation through silencing targets inhibiting ERK, leading to increased ERK phosphorylation. TINCR lncRNA was found to upregulate *BRAF*, leading to increased MAPKC activity. NORAD upregulates ERK1, also leading to increased activity of the MAPKC, resulting in altered proliferation, while BC087858 upregulates MEK, resulting in altered TKI sensitivity. (**D**) TME induction of the MAPK cascade in NSCLC cells through different signaling molecules.

Parallel targeting of MAPKs has been previously assessed in nasopharyngeal carcinoma and bone and soft tissue sarcomas, and preliminary evidence showed promising results [19,20]. Past research also suggested that combined inhibition of the ERK and p38 MAPK pathways (using PD98059 and SB203580) reduced the inflammatory response in monocytes exposed to tumor necrosis factor-alpha [130]. Nonetheless, it is recommended to practice parallel targeting MAPKs cautiously, as different isoforms may have opposing functions that may illicit more severe side effects if targeted incorrectly. This calls for a more comprehensive assessment of the MAPKC [20]. Previous research has documented that ERK inhibition alone could affect the JNK and p38 MAPK pathways in NSCLC, which may lead to paradoxical tumor proliferation [125,131]. Figure 3A shows the possible parallel targets in the MAPKC, with example combinations that could improve treatment response.

It is important to note that while older MAPKCIs had a broader spectrum of activity, impacting the different layers of the MAPKC, novel MAPKCIs have shown a high selectivity profile for the molecular targets in the MAPKC [10]. Older MAPKCIs would, therefore, affect parallel pathways in the MAPKC across different layers. With the paradoxical responses exhibited by downstream components, assessing their effects accurately did not prove easy [28]. Given the heterogeneous genetic profile of NSCLC tumors, the interconnectivity of the MAPKC, and the relevance of other pathways in MAPKCI response, further research should assess the selective combinations of MAPKCIs [125,131,132].

Although current evidence regarding parallel targeting of the MAPKC in NSCLC is scarce, future research should investigate the combinations of MAPKCIs and assess the results of expression of the genes encoding for the kinases in the pathway. A pan-cancer analysis revealed that several isoforms of MAPKs were upregulated in various cancers, including NSCLC [61]. The results of this study not only reinforced the idea that assessing MAPKCIs’ efficacy through evaluating the phosphorylation of the components is insufficient but also indicated the importance of assessing the efficacy of combinations targeting parallel kinases in the MAPKC [61].

### 5.2. Aggregate Targeting of the MAPKC

This review defines aggregate targeting of the MAPKC as any selective MAPKC inhibitor (MAPKCI) combinations that may act on one pathway within the MAPKC. A common practice in testing drug combinations is targeting the RAF/MEK/ERK signaling cascade in NSCLC, the most characterized pathway within the MAPKC [133,134]. Dabrafenib and trametinib, for example, are commonly used to inhibit multiple targets within the RAF/MEK/ERK pathway, particularly RAF and MEK [22].

The significance of aggregate inhibition of a specific pathway is due to the particular paradoxical responses several components within the MAPKC may exhibit. For example, it has been established that using vemurafenib to inhibit ERK would eventually result in a rapid recovery of ERK functions in *BRAF*-mutant NSCLC cells, likely due to the inhibition of ERK-dependent negative feedback [135]. However, using dabrafenib in combination with trametinib to inhibit multiple targets yielded more optimal results in *BRAF*-mutant NSCLC, which led to the approval of this combination [22]. The combination has been considered to change the adverse event profile, yet the underlying mechanisms behind these alterations in the pharmacological activity when using MAPKCI combinations remain unknown [136]. Figure 3B illustrates target combinations for aggregate targeting of the MAPKC.

Past research assessing the concurrent inhibition of the PI3K/Akt and MKK4/JNK pathways revealed synergistic effects on the NSCLC cell lines, although the study did not use highly selective JNK inhibitors [137]. However, the study was conducted in hepatocellular carcinoma cell lines; these findings should be further investigated in NSCLC [138]. With the emergence of MKK7 and MKK4 inhibitors (upstream targets of JNK1/2), it is crucial to investigate the effects of aggregate targeting of this pathway in NSCLC, as it could provide more understanding of this pathway [23,139].

The current literature also lacks studies assessing aggregate inhibition of the p38 MAPK pathway in NSCLC cell lines. While p38 MAPK activation has been considered relevant in chemoresistance in gliomas, combinations of this pathway have not been thoroughly assessed when used in aggregate targeting of p38 MAPK in NSCLC [140]. Novel inhibitors of upstream regulators of p38 MAPK (such as MKK3 and MKK6) have not been used to evaluate aggregate inhibition in NSCLC in addition to p38 MAPK inhibition [128]. Future investigations need to address this critical gap in the literature.

### 5.3. NcRNA Targeting for Optimized Inhibition of the MAPKC

ncRNAs are molecules that do not code for proteins yet are crucial in regulating gene expression and are categorized into short ncRNAs (shorter than 200 nucleotides) and lncRNAs, which are larger than the mentioned threshold [141]. Short ncRNAs are further categorized into microRNAs (miRNAs), small interfering RNAs (siRNAs), and PIWI-interacting RNAs (piRNAs), while lncRNAs include long intergenic ncRNAs (lincRNAs) and circular RNAs (circRNAs), among others [142]. ncRNAs have garnered much attention for their roles in cancer theranostics in recent years, especially since they are more sensitive and specific than other biomarkers for early cancer diagnosis [141,143].

Of the short RNAs, miRNAs are master regulators of cellular functions, with an integral part in cancer proliferation, progression, and metastasis [144]. Several miRNAs have been found to play a significant role in regulating MAPKC components through complex mechanisms. Multiple miRNAs were identified to regulate p38 MAPK through the Nrf2 protein, resulting in an altered therapeutic response [145]. JNKs were also found to be activated by several miRNAs through silencing tumor suppressors, promoting tumor proliferation in various cancers, including NSCLC [146,147]. Moreover, the tumor suppressor miR-16 was downregulated in TKI-resistant NSCLC, as research indicated that miR-16 silenced the expression of the RAF/MEK/ERK pathway components [148]. Conversely, miR-31-5p promotes NSCLC metastasis by silencing targets inhibiting ERK phosphorylation, leading to ERK activation [149].

Targeting miRNAs could potentially reduce drug resistance and increase the treatment efficacy of MAPKCIs, as several in vitro and in vivo experiments established the validity of this approach [150]. Several modalities have been designed to target miRNAs in treating NSCLC, discussed elsewhere [151]. Future literature must address the efficacy of MAPKCIs in combination with miRNA-based therapies and evaluate the predictive value of MAPKC-related miRNAs in NSCLC patients undergoing MAPKCI therapy.

A growing body of literature has also been focused on dissecting the roles and therapeutic and prognostic potentials of lncRNAs in several diseases, including cancer [152]. For NSCLC, lncRNAs were implicated in the tumor’s proliferation, invasion, and metastasis [153]. Mainly, TINCR lncRNA was found to interact with *BRAF* and facilitate NSCLC progression [154]. NORAD is another lncRNA that was found to increase *PEAK1* expression and thus activate ERK [155]. Additionally, lncRNAs were frequently implicated in treatment resistance, making them an attractive drug target for the current research [107]. BC087858 lncRNA induced EGFR-TKI resistance by activating MEK/ERK pathways in NSCLC subpopulations by upregulating *ZEB1* and *Snail* [156]. Figure 3C shows examples of how several miRNAs and lncRNAs affect the ERK cascade.

Recent research has suggested several lncRNAs as factors that could further improve prognosis prediction for immunotherapy [157,158]. Variations in the expression and polymorphisms of lncRNAs were proposed to play a pivotal part in therapeutic responses in NSCLC [159]. Our lab previously showed that MAPK-related lncRNAs could exhibit altered expression under chemotherapy, with several showing paradoxical reactions likely to be involved in tumor proliferation, imposing a risk of aggressive NSCLC recurrence [160]. However, these findings are yet to be verified in vivo. Multiple investigations revealed several lncRNA signatures that have altered expression in post-MAPKCI-treated NSCLC cells [81,161,162,163].

Taken together, these reports suggest a highly relevant role of lncRNAs in NSCLC, and with several lncRNAs having high interplay with the MAPKC, future research must evaluate the effects of MAPKCIs on lncRNA alterations [106]. Moreover, expression changes in MAPK-related lncRNAs may prove helpful prognostic markers in the response to MAPKCIs, which also requires further investigation. Incorporating lncRNA targeting into parallel and aggregate MAPKC targeting may improve NSCLC patients’ outcomes. While RNA-based therapeutics face several challenges in their application, recent developments in drug-delivery nanotechnology have shown promising results, which may improve their implementation in treating NSCLC patients [164].

### 5.4. Tumor Microenvironment (TME) Considerations in Navigating Drug Resistance

Qin et al. conducted a study to map intrinsic and extrinsic cell signaling to assess the effects of oncogenic mutations and TME signals on colonic organoids [165]. The study identified the TME-related induction of oncogenesis by MAPK activation through EGF ligands secreted by TME fibroblasts [165]. Studies have also identified many other mechanisms through which the TME would contribute to increased tumorigenesis by activating the MAPKC in NSCLC [166,167,168,169]. Interestingly, MAPK activation was also recently shown to confer EGFR inhibitor resistance in NSCLC through TME modulation [166].

Other recent studies have identified that newly discovered T-cell subsets, known as Th22 cells, were implicated in NSCLC regulation through interleukin (IL)-22 [167]. Th22 cells were found to have elevated secretion of IL-22 in tumoral tissues, which induced drug resistance, promoted cancer cell migration, and inhibited cell cycle arrest in NSCLC cells [167,168]. Peritumoral tissue of NSCLC was also found to play a part in further induction of the MAPKC through cytochrome P450 2E1 (CYP2E1) [169]. CYP2E1 is hypothesized to be associated with higher activation of ERK1/2 and p38 MAPK in NSCLC and peritumoral tissues [169]. Figure 3D illustrates the possible mechanisms through which the MAPKC can be activated from TME-related modulation in NSCLC.

Alterations in the TME associated with MAPKC activation have been shown to have more pronounced effects on the immune TME, affecting programmed cell death-ligand 1 (PD-L1) expression in NSCLC cells [170]. A study assessing the inhibition of PD-L1 with a MAPKCI in NSCLC organoids revealed that the therapeutic combination showed synergistic efficacy [171]. While not all combinations of MAPKCIs and immune checkpoint inhibitors have synergistic effects, future research should investigate these combinations in the context of parallel and aggregate targeting of the MAPKC and TME in NSCLC [16].

The involvement of the MAPKC in TME modulation urges a more comprehensive evaluation of this pathway when targeted with selective inhibitors and other therapeutic modalities, particularly in NSCLC. One emerging therapeutic approach that should be investigated for targeting the TME in combination with MAPKCIs is bacterial immunotherapy [172]. Studies showing promising effects of novel methods utilizing bacteria for their antitumor efficacy and concerns translating the findings of the in vitro studies to clinical settings warrant further investigation of the MAPKC effects on these treatments [173,174].

## 6. Discussion

Despite the broad literature available on this pathway, the MAPKC is a complex signaling pathway and a pivotal regulator of various cellular processes, which makes its ultimate characterization difficult [175]. More importantly, research often focuses on commonly mutated effectors of the MAPKC, such as EGFR, KRAS, MET, and ROS1, due to the prevalence of these mutations and the lack of adequate characterization of the MAPKC [4]. Despite the rigorous developments in targeted therapies, the results remain underwhelming for NSCLC patients, attributed to the genomic instability of these tumors [116].

The research focus on EGFR, KRAS, and other MAPKC upstream effectors came at the cost of a scarcity of downstream MAPKC component assessments [117]. While mutations in the MAPKC are considered rare, a recent investigation indicated that alterations in the expression of genes encoding for these components are often overlooked [61]. Research also regards the current methods of assessing the efficacy of used MAPKCIs as inaccurate; thus, further improvements are highly recommended [25,60].

Recently, multiple assessment modalities of MAPKC activity have been found to indicate MAPKC activity compared to the phosphorylation of MAPKC components. The MAPK Pathway Activity Score (MPAS), a modality of evaluating MAPKC activity through the aggregated expression of a panel of 10 MAPKC target genes, was more accurate in reporting MAPKC inhibition when using specific inhibitors [101,176]. A study found that the MPAS was suboptimal in predicting BRAF and ERK inhibitor sensitivity and was more accurate in predicting MEK inhibitor sensitivity [25]. The research by Sigaud et al. has identified MAPKCI sensitivity scores (MSSs) as predictive of inhibitor sensitivity driven by immune infiltration in vitro and in vivo in pediatric gliomas [25]. The authors report that the MPAS and MSSs may have different utilities, with the MPAS assessing MAPK activation and MSSs predicting sensitivity. These results are yet to be validated in NSLC clinical trials.

While in vitro studies of MAPKCIs on NSCLC cell lines provided promising results, their efficacy lacked reproducibility in some patient populations in clinical trials, indicating the need for a more robust characterization of this pathway [177]. Additionally, clinical trials assessing the efficacies of MAPKCIs in NSCLC patient subpopulations according to their mutational profiles often report a small sample size [109,111,114]. This should be addressed in future, more extensive clinical trials. A comprehensive understanding of the mutational background of patients receiving MAPKCIs may provide a more cohesive picture of which subpopulations may respond better to specific treatments. With the recent patents of novel MAPKCIs, future research should be able to perform more accurate assessments using MAPKCIs with higher selectivity profiles [10].

A gap the current literature has not adequately addressed is the efficacy of parallel targeting of the p38 MAPK and JNK signaling pathways in NSCLC using MAPKCI combinations, which should be investigated in future research. Moreover, the lack of assessing combinations of MAPKCIs for aggregate targeting of the MAPKC should also be addressed in NSCLC, as recent studies have already revealed promising results for p38 MAPK and JNK inhibitors in other cancers [128,178].

Assessing these combinations and their effects on the genetic expression of MAPKC components and MAPKC-related ncRNAs may provide insights into which NSCLC patient subpopulations are more vulnerable to MAPKCIs [86]. Drug developments made many of the components in the MAPKC targetable, and many of these targets have been shown to provide impressive results. Yet, no studies have assessed such combinations (such as MLK/MKK/JNK inhibition) in NSCLC [179].

Advances in research evaluating prognostic factors in the TME may provide further indications for studies incorporating MAPKCIs and bacterial therapeutic combinations [172]. It remains challenging to conclude bacterial immunotherapy’s usefulness and clinical efficacy as monotherapy. Combining MAPKCIs that interact with the NSCLC TME may enhance the efficacy of bacterial immunotherapy, although thorough investigations of these combinations need to be performed.

## 7. Conclusions

A broad understanding of the MAPKC may provide insight into vulnerable NSCLC patient groups. While MAPKCIs have shown promising results, further research must be conducted on combining these targeted therapies in NSCLC. In this review, we suggest two particular approaches, parallel targeting and aggregate targeting of the MAPKC, which could enhance the combinatorial efficacy of selective MAPKCIs that have shown effectiveness in other cancers but have yet to be comprehensively assessed in NSCLC.

As the current literature often evaluates the efficacies of MAPKCI combinations and other therapies through the phosphorylation of the MAPKC components, alternative assessment methods should be used. Alterations in genetic expression for genes encoding MAPKC kinases and regulators need more rigorous investigations, in addition to the changes incurred to genetic expression through MAPKCIs.

Future research studies should consider the gap in the literature regarding the investigation of different combinations of selective inhibitors for the p38 MAPK and JNK pathways in NSCLC. The lack of evidence on these targets limits our understanding of the MAPKC, as it is often only targeted through the RAS/RAF/MEK/ERK signaling cascade, leaving much more to be further explored. With many novel agents developed to target the p38 MAPK and JNK pathways selectively, assessments of the efficacy of parallel and aggregate targeting of the MAPKC should also be addressed in future research.

Nonetheless, variations in ncRNAs regulating the MAPKC could also be a promising research topic, as they may provide insights regarding the inconsistent response rates to MAPKCIs in NSCLC patients. As the research on ncRNAs extends, especially regarding their pivotal role in regulating the MAPKC and its crosstalk, novel approaches to reduce the associated drug sensitivity might be developed along the way.

## 8. Future Directions

Future research should assess alterations in gene expression for gene targets of the MAPKC when using MAPKCIs, in addition to the phosphorylation of these components, for a more comprehensive assessment of the changes sustained by this cascade. Further, studies should include a more refined methodology regarding the use of drug targets when assessing MAPKCIs in NSCLC in vitro. Incorporating parallel or aggregate inhibition of the MAPKC may improve the effectiveness of its inhibitors, providing a clearer understanding of MAPKC components and their function, particularly of p38 and JNK.

Combining MAPKCIs with other therapeutic agents in treating tumors should be addressed considering the alterations in MAPKC-related ncRNA expression and the expression of the genes encoding for MAPKC components. Future clinical trials should include an assessment of these alterations rather than exclusively relying on the phosphorylation of MAPKC kinases to assess the activity of the MAPKCIs.

## Figures and Tables

**Table 1:** Selected in vitro studies investigating some of the effects of MAPK pathway inhibitors in NSCLC.

MAPK Pathway Inhibitor	MAPKC Targeted Molecules	In Vitro Model	Genomic Profile Alterations	Effect	References
1195765-45-7 *Dabrafenib*	RAF	Human lung adenocarcinoma cell lines PC9 and HCC827 and EGFR TKI-resistant human lung adenocarcinoma cell lines PC9ZD and HCC827GR55	*EGFR* HCC827 is an exon 19 EGFR-mutated cell line HCC827GR55 is an MET-amplified cell line	Dabrafenib reduced ERK and AKT phosphorylation	[70]
		HEK293T cells were used as well as mouse-derived lung tissue organoids	*BRAF^V600E^* and *EGFR^L858R^*	Dabrafenib and trametinib combination inhibited BRAF^V600E^ signaling, reducing ERK1/2 phosphorylation	[69]
		Lung cancer cell lines (A-427, A549, Calu-1, Calu-3, HCC827, NCI-H460, and NCI-H1299); lung cancer stem cell line; pancreas (HPDE, MiaPaCa2, T3M4, PANC1, PACA44, HPAFII, PT45P1, and L3 6p1); colon (HCT116, HK2-6, and HKE-3); and organoids (N1, T1, T5, T9, and T14) were used	*BRAF* and *KRAS*	*KRAS* mutation is not the sole predictor of therapeutic synergism of RAF and MEK inhibitors; RAF inhibition with dabrafenib alone induced paradoxical MAPK activation through increased ERK phosphorylation; combination with trametinib reduced ERK phosphorylation synergizing with dabrafenib	[91]
918504-65-1 *Vemurafenib*	RAF	Lung adenocarcinoma PDX model (PHLC12) and a PDX-derived cell line (X12CL)	*BRAF^G469V^*	BRAF protein kinase activity was inhibited	[71]
		The NSCLC cell line used was NCI-H2122; other cell lines used for melanoma and pancreatic cancer were BxPC3, Panc1, MiaPaca2, BxPC3M1, and SK-MEL-28	N/A	Vemurafenib sensitized the NSCLC cell line to gemcitabine	[72]
		NSCLC cell lines A549, H460, H1755, and HCC364	*BRAF^V600^* and *BRAF^G469A^*	Vemurafenib and erlotinib were not synergistic in inhibiting p-ERK; vemurafenib and trametinib showed stronger efficacy than either single agent alone	[92]
606143-89-9 *Binimetinib*	MEK1/2	H596, EKVX, H1975, HCC827, Calu-1, H1792, H332M, H358, H23, H460, A549, H1299, H522, and H157 NSCLC cell lines	*KRAS*, p53, *PTEN*, *EGFR*, *LKB1*, *PIK3CA*, and *CDKN2A*	Binimetinib reduced cyclin D1 protein expression in sensitive cells, arresting cell lines in the G1 phase	[73]
		H1975, H460, and A549 NSCLC cell lines	*EGFR*, *KRAS*, *PIK3CA*, and *MET*	Concurrent targeting of MEK and PI3K pathways inhibited proliferation and decreased phosphorylation of ERK1/2; cells with *KRAS* and *PIK3CA* mutations were most sensitive to MEK162 (binimetinib) and BKM120 combination	[93]
934660-93-2 *Cobimetinib*	MEK1/2	H460, A549, and H1975 cell lines	*PIK3CA*, *KRAS*, *IDH1*, *CDKN2A*, *EGFR*, and *PIK3R1*	GDC-0973 (cobimetinib) reduced ERK phosphorylation	[75]
		MOR, NCI-H2122, A549, and NCI-H441 NSCLC cell lines	MOR cells were *KRAS* mutant	Cobimetinib enhanced MAPK7 phosphorylation; combinations with other MAPKCIs potentiated effects of cobimetinib	[76]
871700-17-3 *Trametinib*	MEK1/2	Patient-derived xenograft mouse models and cell lines including H460, Calu-6, H441, H292, A549, H23, H1944, and H1358 and trametinib resistant A549 and H23 cell lines	PDX (*KRAS^G12V^* and *KRAS^G12C^* mutated) cell lines had various *KRAS* mutations (H460 cell line had the *KRAS^Q61H^* mutation; Calu-1, *KRAS^G12C^*; H441, *KRAS^G12V^*; H292, *KRAS^G12S^*; A549, *KRAS^G12S^*; H23, *KRAS^G12C^*; H1944, *KRAS^G12C^*; and H358, *KRAS^G12C^*)	MEK inhibition upregulated PDP2 and downregulated PDHK1 expression in trametinib-resistant cell lines; trametinib further enhanced ROS-responsive expression in trametinib-resistant cells	[79]
		A549 and H460 cells	N/A	A combination of trametinib and BC-LI-0186 inhibited the MTORC1-activating function of LARS1 in NSCLC with no apparent treatment target mutations	[80]
		PC9 and HCC827 NSCLC cell lines	*EGFR* and *BRAF^G469A^*	Osimertinib-resistant cells were resensitized by combining osimertinib and trametinib (but not dabrafenib); the combination exerted significant effects on *BRAF*^nonV600E-mutated^ cell lines	[94]
		NSCLC cell lines A247, CALU-3, NCI-H1299, NCI-H1693, NCI-H1838, NCI-H2087, NCI-H2170, NCI-H292, NCI-H358, and NCI-H460	*KRAS* and *NRAS*	ERK was not a sufficient predictor for trametinib response; trametinib induces its cytotoxic effects depending upon cellular context more than oncogenic status, and combination therapy with other parallel pathway inhibitors may confer improved responses especially in *BRAF* wild-type NSCLC	[95]
1236699-92-5 *Pimasertib*	ERK1/2	Human *KRAS* mutant CRC cell lines (HCT116, GP5d, LS174T, SK-CO-1, LoVo, SW948, H747, SW837, LS1034, T84, and SW1116, and human KRAS mutant NSCLC cell lines (DV-90, A427, A549, H460, H1944, LU-99A, SW1573, H1792, H2030, COR-L23, H358, and Calu-6)	*KRAS*	Pimasertib upregulated genes regulated by p53 (CDKN1A and MDM2) and downregulated genes regulated by ERK (DUSP6, SPRY4, and ETV4)	[81]
		Human COLO205, HT29, LOVO, and HCT15 colorectal cancer cell lines and H1299, A549, H460, and H1975 NSCLC cell lines; mice bearing subcutaneous tumor xenograft of HCT15 and H1975 cell lines were also established	*KRAS*, *NRAS*, *BRAF*, *PIK3CA*, and *EGFR*	No direct correlation was found between mutational status and cell line sensitivity to pimasertib; combinations with other pathway inhibitors (mTOR, PI3K) and with multi-kinase inhibitors resulted in synergistic effects	[96]
152121-30-7 *SB202190*	ERK1/2, p38 MAPK	A549, H226, and H1299 NSCLC cell lines	H226 cells were p53-mutated	SB202190 reduced p38 MAPK, ERK1/2, and AKT^S473^ phosphorylation	[83]
		Ba/F3 cells	*CD74-ROS1* fusion, *ROS1*^F2004V^, and *ROS1*^F2075C^ mutations	SB202190 inhibited p38 MAPK signaling	[84]
		NSCLC cell lines H520 and H1703	N/A	SB202190 elevated the cytotoxic effects of pemetrexed on NSCLC cell lines through inhibiting p38 MAPK and reducing its phosphorylation	[97]
		NSCLC cell lines A549 and NCI-H358	N/A	Apoptotic effects exerted by matrine on NSCLC cell lines were partially reduced by inhibition of p38 MAPK through SB202190	[98]
		NSCLC cell lines H1975 and A549	N/A	Etoposide increased MKK3/6 and p38 MAPK phosphorylated protein levels; inhibiting p38 MAPK with SB202190 sensitized NSCLC cells to etoposide	[99]
464-92-6 *Asiatic Acid*	P38 MAPK and JNK	A549/DDP cells	MALAT1	AA downregulated MALAT1, p300, β-catenin, and MDR1; AA upregulated miR-1297 expression	[86]
		A549 cells	N/A	AA upregulated miR-1290 and downregulated BCL2 expression	[87]
585543-15-3 *Losmapimod*	P38 MAPK	HCC827, H1975, HEK293T, and HCC827GR cells	*EGFR*	Losmapimod reduced p38 MAPK and STAT3 phosphorylation and downregulated p21 and cyclin D1	[88]

N/A: Not available/ not reported.

**Table 2:** Clinical trials on the effects of MAPK pathway inhibitors in NSCLC were recently completed.

MAPK Pathway Inhibitor	Main MAPKC Targeted Molecule	Name	Participants	Mutations	Condition	Aims	Outcomes	Identifier
1195765-45-7 *Dabrafenib*	RAF	A Phase II Study of the BRAF Inhibitor Dabrafenib as a Single Agent and in Combination With the MEK Inhibitor Trametinib in Subjects With *BRAF^V600E^* Mutation Positive Metastatic (Stage IV) Non-small Cell Lung Cancer	177	*BRAF^V600E^*	NSCLC	To assess dabrafenib and trametinib efficacy safety and tolerability	ORR in cohort A (dabrafenib monotherapy) was 33% and PFS was 5.5; ORR in B (combined treatments and treatment-naïve cohort) and C (pretreated) cohorts was 68.4% and 63.9%; median PFS was 10.2 and 10.8; OS was 18.2 and 17.3; 5-year OS was 34% and 22%	NCT01336634
606143-89-9 *Binimetinib*	MEK1/2	A Phase II, Open Label, Multiple Arm Study of Single Agent AUY922, BYL719, INC280, LDK378, and MEK162 in Chinese Patients With Advanced Non-small Cell Lung Cancer (NSCLC)	66	*KRAS*, *NRAS*, and *BRAF*	NSCLC	To assess the anti-tumor activity of alpelisib, capmatinib, ceritinib, and binimetinib	Partial responses: alpelisib arm was 0%; capmatinib was18.8%; ceritinib was 73.1%; binimetinib was 9.1%	NCT02276027
934660-93-2 *Cobimetinib*	MEK1/2	A Study of the Safety, Tolerability, and Effects of Cobimetinib and GDC-0994 in Patients With Locally Advanced or Metastatic Solid Tumors	24	N/A	Multiple cancers, including NSCLC	To evaluate safety, tolerability, and clinical effects of cobimetinib, and ravoxertinib	Daily treatment cohort required a reduction in cobimetinib dose due to grade 1–2 toxicity; intermittent schedule treatment cohort dose increases for both treatments were intolerable due to grade 3 toxicity of myocardial infarction; 7 patients achieved stable disease	NCT02457793
		Study of Atezolizumab in Combination With Cobimetinib in Participants With Locally Advanced or Metastatic Solid Tumors	150	*KRAS* and *BRAF*	NSCLC, colorectal cancer, melanoma, other solid tumors	To investigate safety and activity of combining MEK inhibition with PD-L1 inhibition	The combination had tolerable safety and clinical activity; ORR was in 18% of NSCLC patients	NCT01988896
871700-17-3 *Trametinib*	MEK1/2	A Phase II Study of the BRAF Inhibitor Dabrafenib as a Single Agent and in Combination With the MEK Inhibitor Trametinib in Subjects With *BRAF^V600E^* Mutation Positive Metastatic (Stage IV) Non-small Cell Lung Cancer	117	*BRAF^V600E^*	NSCLC	To assess dabrafenib and trametinib efficacy safety and tolerability	ORR in cohort A (dabrafenib monotherapy) was 33% and PFS was 5.5; ORR in B (combined treatments, treatment-naïve cohort) and C (pretreated) cohorts was 68.4% and 63.9%; median PFS was 10.2 and 10.8; OS was 18.2 and 17.3; 5-year OS was 34% and 22%	NCT01336634
		Trial of Trametinib and Ponatinib in Patients With KRAS Mutant Advanced Non-Small Cell Lung Cancer	12	*KRAS*	NSCLC	To assess combination of trametinib and ponatinib efficacy, tolerability, and safety	No response was observed	NCT03704688
923032-37-5 *Refametinib*	MEK1/2	A Phase 1/2 Study of the Combination of RDEA119 and Sorafenib in Patients With Advanced Cancer	62	*KRAS*, *BRAF*, and *PIK3CA*	Advanced cancer	To assess safety and tolerability of refametinib in combination with sorafenib	Disease was stabilized in approximately half the patients	NCT00785226
1236699-92-5 *Pimasertib*	ERK1/2	An Open-Label, Phase Ib Dose Escalation Trial of Oral Combination Therapy With MSC1936369B and SAR245409 in Subjects With Locally Advanced or Metastatic Solid Tumors	146	*PTEN*, *BRAF*, *KRAS*, *NRAS*, *PI3KCA*, *EGFR*, *ERBB2*, *MET*, *RET*, *c-KIT*, *GNAQ*, and *GNA11*	Multiple cancers, including NSCLC	To assess safety, efficacy, pharmacodynamics, and kinetics as well as maximum tolerated dose of pimasertib and voxtalisib	Combination achieved stable disease in 46% but was not tolerable as a long-term treatment	NCT01390818
1453848-26-4 *Ravoxertinib*	ERK1/2	A Study of the Safety, Tolerability, and Effects of Cobimetinib and GDC-0994 in Patients With Locally Advanced or Metastatic Solid Tumors	24	N/A	Multiple cancers, including NSCLC	To evaluate safety, tolerability, and clinical effects of cobimetinib and ravoxertinib	Daily treatment cohort required a reduction in cobimetinib dose due to grade 1-2 toxicity; intermittent schedule treatment cohort dose increases for both treatments were intolerable due to grade 3 toxicity of myocardial infarction; 7 patients achieved stable disease	NCT02457793

PFS: progression-free survival, expressed as median in months; OS: overall survival rate; ORR: objective response rate; N/A: Not available/ not reported.
